# Human sex recognition: a developmental voice-based model of brain circuits

**DOI:** 10.3389/fnbeh.2026.1714154

**Published:** 2026-05-29

**Authors:** Yehuda Salu

**Affiliations:** Department of Physics and Astronomy, Howard University, Washington, DC, United States

**Keywords:** auditory processing, development, human sex recognition, limbic–hypothalamic system, medial geniculate nucleus, multimodal integration, sensory substitution, voice perception

## Abstract

Pheromonal, neuroimaging, and comparative evidence converge on a limbic–hypothalamic axis—including the amygdala, bed nucleus of the stria terminalis (BNST), and hypothalamus—that organizes sex-related behaviors. In many mammals, this system is driven by chemosensory input via the vomeronasal organ (VNO) and accessory olfactory bulb (AOB). In humans, however, the functional role of the VNO is uncertain, and no consistent sex pheromone has been identified. The Human Sex Recognition (HSR) model proposes a sensory substitution at the level of input: auditory signals, conveyed through the medial geniculate nucleus (MGN), provide the primary entry point into this conserved system. Within this framework, human voice signals are proposed to serve as a functional analog to pheromonal cues in other mammals. Developmentally, auditory cues, available prenatally and throughout childhood, provide an early scaffold for the acquisition of visual and other sex-related cues through cortical learning. These multimodal representations converge on limbic–hypothalamic circuits, which are reorganized at puberty to support the participation of sex recognition in sex-related behavior. By specifying the primary sensory channel interfacing with a conserved neural architecture, the HSR model offers a biologically grounded alternative to pheromone-based accounts and generates testable predictions regarding the neural and developmental basis of human sex recognition.

## 1 Introduction

Sex recognition is an important component of many social and sex-related behaviors. In this context, “sex recognition” refers to the process by which another individual is categorized as male or female based on available sensory information. This process contributes not only to sex related behaviors, but also to broader social behaviors such as communication, social categorization, and group interaction. Within the HSR framework, sex recognition is treated as an early component of sex-related behavioral systems, relying on both innate and learned cues, including auditory, visual, and contextual signals acquired through development.

Across many species, sociosexual related behavior begins developing before puberty through the progressive association of sensory, emotional, motivational, and motor cues into coordinated behavioral programs (Dulac and Torello, 2003; Pellis and Pellis, 2009; Schulz et al., 2009). During this period, immature organisms associate social cues with emotional responses, interaction patterns, and biologically relevant sex-related signals. In rodents, early sensory and social experience can influence later mate preference and sociosexual related behavior, including responses to auditory and chemosensory cues (Asaba et al., 2014). Puberty does not create these systems *de novo*, but rather reorganizes and activates previously developing limbic–hypothalamic circuits, enabling mature sex related behavior (Sisk and Zehr, 2005; Schulz et al., 2009).

In rodents, pheromonal cues are thought to play a major developmental role in organizing these associations. The HSR model proposes that auditory cues assume a comparable scaffolding function in humans. Specifically, voice is proposed to serve as an early and prominent cue through which sex-related social associations are progressively organized. Through repeated experience, auditory cues become linked with visual appearance, emotional responses, social context, and behavioral expectations, gradually forming multimodal representations that later contribute to adult sex recognition and sex related behaviors.

In many mammals, pheromones are detected by the vomeronasal organ (VNO) and drive sex related behaviors through pathways involving the accessory olfactory bulb (AOB), amygdala, BNST, and hypothalamus (Dulac and Torello, 2003). Pheromone-based models have also been proposed for humans (Savic et al., 2001; Jacob et al., 2002). However, no specific human sex pheromone has been identified, the human VNO is vestigial or functionally uncertain (Meredith, 2001), and the available chemosensory evidence remains inconsistent. These limitations raise the question of whether another sensory channel provides the primary developmental input to human sex-recognition systems.

Developmental and neuroimaging findings point to the human voice as a strong candidate. Infants exhibit early specialization for human voice processing, while reliable audiovisual matching of sex-related facial and vocal cues emerges later in development (Grossmann et al., 2010; Richoz et al., 2017).

Children progressively integrate voices with visual recognition (Campanella and Belin, 2007), and puberty reorganizes hypothalamic–limbic circuits involved in sexual motivation (Sisk and Zehr, 2005). These findings suggest a developmental sequence in which auditory cues provide an early scaffold, visual and contextual cues are incorporated through learning, and puberty enables the resulting multimodal representations to participate in sex related behavior.

The HSR model represents the most recent iteration of a framework developed over more than a decade. Its first version (Salu, 2011) proposed that voice functions as a primary innate cue anchoring associations with other sensory inputs, including visual signals, and emphasized prenatal influences. A later refinement (Salu, 2020) elaborated the role of subcortical circuits comprising the medial geniculate nucleus (MGN), amygdala, BNST, and hypothalamus. The present formulation extends these earlier proposals in three ways: it organizes the model into an explicit developmental trajectory from prenatal auditory sensitivity to pubertal activation; it proposes a sensory substitution principle in which auditory input via the MGN functionally replaces vomeronasal input as the primary entry point into a conserved limbic–hypothalamic architecture; and it distinguishes well-supported indirect cortical–limbic routes from more speculative direct or subcortical pathways.

The HSR model does not propose a fundamentally new organizational framework for sex recognition. Rather, it builds on the broader cortical–limbic–hypothalamic architecture already implicated in social and sex related behavior. In this framework, the proposed substitution occurs at the level of sensory input: auditory signals, conveyed through the medial geniculate nucleus (MGN; also referred to as the medial geniculate body, MGB), are proposed to provide the primary entry point into a system that, in other mammals, is often driven by chemosensory input through the VNO/AOB pathway.

This broader architecture includes cortical regions that process socially relevant information and modulate subcortical responses. Auditory and visual association areas contribute to identity recognition; superior temporal regions process voices and biological motion; and higher-order cortical areas, including prefrontal and orbitofrontal cortices, integrate contextual, social, and motivational information. These systems provide information about the physical characteristics, identity, and social context of other individuals and shape the responses of limbic and hypothalamic circuits.

Empirical studies have identified sex-differentiated processing of both auditory and visual social cues in humans. Neuroimaging studies have reported differential cortical and subcortical responses to male and female voices, including sex-dependent activation patterns in auditory cortex, amygdala, and hypothalamic regions. Similarly, visual sexual stimuli elicit limbic and hypothalamic responses that vary with stimulus type, biological sex, and sexual orientation (Stoléru et al., 2012). The HSR model does not interpret these findings as evidence for fully separate male and female anatomical pathways. Instead, it proposes that developmentally acquired auditory–visual associations help organize how multimodal sex-related cues gain access to conserved limbic–hypothalamic circuitry.

Within this architecture, auditory input may influence hypothalamic sex-related centers through both indirect and more direct routes. Indirect pathways are better supported and likely involve auditory cortex, association areas, and limbic structures such as the amygdala, which in turn connect to the hypothalamus. Shorter subcortical routes from the MGN, particularly its medial division, to limbic structures may also contribute to rapid processing of biologically relevant sounds. More direct auditory–hypothalamic pathways remain less established in humans and are presented here as testable hypotheses of the HSR model.

**Figure 1:**
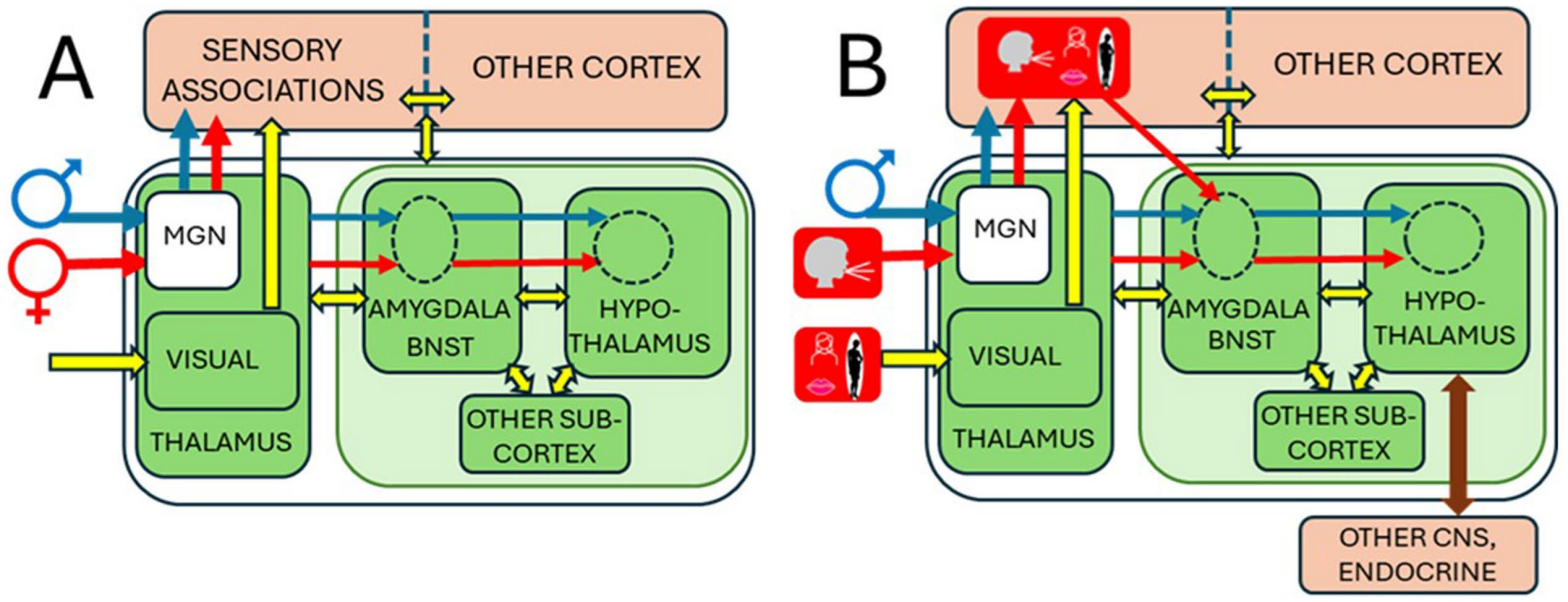
The human sex recognition (HSR) model. The figure illustrates functional information pathways proposed by the HSR model. The main difference between the HSR model and pheromone-based models lies in the proposed source of the primary innate sex-recognition cues. In pheromone-based models, chemosensory cues enter the system through the vomeronasal organ (VNO) and accessory olfactory bulb (AOB). In the HSR model, male- and female-associated voice cues enter through the auditory system, including the medial geniculate nucleus (MGN), and provide the primary developmental scaffold for learning other sex-recognition cues. **(A)** Innate auditory framework. Thick blue and red arrows indicate empirically supported auditory processing of male- and female-associated voice cues, respectively. Thin blue and red arrows indicate HSR-proposed functional pathways by which male- and female-associated auditory information may influence limbic–hypothalamic sex-recognition systems. Yellow arrows indicate other empirically supported functional pathways within the broader cortical–limbic–hypothalamic framework. **(B)** Learning of visual sex-recognition cues. Auditory and visual female cues from the same individual enter the system together. In the HSR model, auditory cues serve as a scaffold for associating visual features with sex-related information. These auditory–visual associations are formed in cortical association areas through established multisensory learning pathways. Learned visual cues may then influence limbic and hypothalamic systems through cortical–subcortical pathways, including projections involving the amygdala and BNST. At puberty, hormonal reorganization of hypothalamic, limbic, CNS, and endocrine interactions enables these learned multimodal cues to participate in sex recognition and sex-related behavior (brown arrow). Similar learning processes are proposed for male-associated visual cues and for other learned cues across sensory modalities.

Accordingly, the present work focuses on the developmental and neural mechanisms underlying sex recognition itself. Sexual attraction, partner preference, and sexual orientation may depend in part on sex recognition, but they also involve additional cognitive, emotional, and social factors beyond the scope of this model. The HSR framework is therefore intended to explain the development and operation of human sex recognition, rather than to provide a comprehensive theory of sex related behavior or orientation (see Figure 1).

## 2 The human sex recognition (HSR) model

The model is based on the following observations:

- *Prenatal foundations*: prenatal auditory exposure provides the initial basis for postnatal processing of human voices. Fetuses demonstrate sensitivity to both maternal and unfamiliar speech rhythms, can discriminate sounds by pitch, and distinguish familiar from unfamiliar voices (Kisilevsky et al., 2003; Kisilevsky et al., 2009). Evidence confirms that the auditory system is functional during late gestation and responsive to external voices (Chelli and Chanoufi, 2008). These findings suggest that the auditory system is primed before birth to process sex-differentiated auditory cues.
  While direct evidence for prenatal sensitivity to sex-differentiated auditory cues is limited, the acoustic differences between male and female voices—particularly in fundamental frequency and timbre—are present in the prenatal environment. Repeated exposure to these differences may contribute to the early organization of auditory processing, which is further refined through postnatal experience. In this sense, prenatal auditory input is best understood as preparing the system for later development of sex recognition, rather than establishing fully formed sex-specific representations.
- *Auditory scaffold in infancy*: immediately after birth, infants show specialized brain responses to voices (Grossmann et al., 2010). By four months, voice-selective processing is evident (Calce et al., 2024), and by 4.5 months infants can distinguish unfamiliar female voices (Yu et al., 2024). These results support the idea that voice processing is not only innate but functionally specialized, supporting early categorization of human vocal signals.
- *Childhood cross-modal learning*: as visual processing matures, cortical regions such as the fusiform face area (FFA), superior temporal sulcus (STS), and occipitotemporal cortex bind visual cues with auditory cues. Infants and children can match voices to faces by gender (Richoz et al., 2017), and by 9–12 months integrate biological motion cues with voices (Johnson et al., 2021). Vocal cues may even outweigh facial cues for certain judgments (Blasi et al., 2023), supporting the formation of multimodal representations associated with sex categories.
- *Pubertal expansion*: at puberty, gonadal hormones reorganize hypothalamic–limbic circuits (Sisk and Zehr, 2005). Voice undergoes striking pubertal changes, sharpening its salience as a sex-differentiated cue (Salu, 2011). These changes are universal and obvious; no sophisticated instrumentation is needed to notice them. Yet, careful acoustic analyses reveal that subtler sex-related differences in voice parameters emerge gradually between the ages of 5 and 15, allowing reliable recognition of a child’s sex from speech alone (Chen et al., 2022). In parallel, adolescents exhibit increased hypothalamic responses to visual sex cues compared to children, reflecting a developmental shift toward visual dominance (Nowling et al., 2024).

To clarify the developmental progression proposed by the HSR model, Table 1 summarizes the major stages from prenatal auditory exposure to pubertal activation. The transitions between stages reflect shifts in both the dominant sensory inputs and the underlying neural organization: from prenatal auditory sensitivity, to postnatal voice-based categorization, to childhood cross-modal integration, and finally to hormonally driven reorganization of hypothalamic–limbic circuits at puberty.

**Table 1:** Developmental progression of human sex recognition in the HSR model.

Stage	Period	Dominant inputs	Developmental role in HSR
Prenatal	Late gestation	voice rhythm, pitch, prosody	Establishes early sensitivity to human voices and sex-related acoustic features
Infancy	Birth – ~1 yr.	Auditory, limited visual	Voices act as a primary scaffold for early categorization of human agents
Childhood	1 yr– puberty	Auditory, full visual	Cross-modal learning links visual and social cues to auditory templates
Puberty	Adolescence	Multimodal (voice + visual)	Hormonal reorganization enables sex recognition to guide sex related behavior

The HSR model integrates empirically established findings—such as fetal auditory sensitivity, early voice-selective processing, and pubertal neuroendocrine changes—with theoretical proposals regarding how these processes are organized into a coherent developmental sequence. In this framework, auditory cues are proposed to function as the primary scaffold for the acquisition and integration of other sex-related cues, while specific pathways linking auditory processing to hypothalamic sex-related centers remain, in part, hypothetical and subject to future empirical testing.

Empirical findings underlying each developmental stage are detailed and referenced in the main text. The organization into stages, dominant inputs, and developmental role reflects the proposed HSR framework. Additional details regarding neural substrates and stage transitions are described in the text.

## 3 Comparison with pheromone-based models

Pheromone models propose that chemosensory cues function as primary sex recognition signals in humans. Evidence includes hypothalamic activation to putative pheromones (Savic et al., 2001; Savic and Lindström, 2008). However, limitations include the absence of a universal human sex pheromone, inconsistent behavioral effects, and unclear developmental roles.

In contrast, the HSR model accounts for early sex recognition (auditory before visual), cross-modal integration during childhood, and puberty-related neural changes, supported by developmental studies of voice and face processing. It should be noted that some authors have suggested that non-VNO chemosensory pathways, via the main olfactory system, may modulate social communication in humans (Herz and Inzlicht, 2002). However, consistent with Trotier’s (2011) conclusion, such influences appear modulatory rather than determinative for human mate choice or sexual recognition.

## 4 Scientific method evaluation

The HSR model is falsifiable, with predictions such as impaired childhood sex recognition in cases of auditory deprivation, whereas pheromone models predict impairment with olfactory deprivation. The HSR model is empirically supported by prenatal voice recognition, infant voice selectivity, and developmental neuroimaging. Although moderately complex, the HSR framework offers a broader set of developmental and neurobiological predictions than pheromone-only models. In addition, the model demonstrates biological feasibility, as discussed in Section 6.1, which strengthens its plausibility compared to pheromone-based accounts.

## 5 Predictions and testable hypotheses

1. Children should classify sex by voice earlier than by face.
2. Congenital deafness should impair childhood sex recognition more than olfactory impairment.
3. Adolescents should show increased hypothalamic activation to visual sex cues compared to children.
4. If pheromone-based models are correct, olfactory input should play a necessary role in human sex recognition. In that case, individuals with impaired olfactory function (e.g., congenital anosmia or acquired smell loss) would be expected to show deficits not only in aspects of sex-related behavior or social interaction, but also in recognizing the sex of other individuals from sensory cues. Existing studies instead suggest that olfactory deficits primarily affect emotional intimacy, partner bonding, sexual activity, and social confidence, rather than basic sex recognition itself (Oleszkiewicz et al., 2020; Croy et al., 2013).

By contrast, the HSR model predicts that olfactory input is not required for sex recognition itself, as this function is primarily supported by auditory and visual cues. Under this framework, individuals with olfactory deficits may exhibit changes in sex-related behavior, emotional responses, or social bonding, but should retain the ability to recognize sex from voice and visual cues.

To date, while olfactory deficits have been associated with reduced sexual activity and altered social relationships, there is no direct evidence that they impair sex recognition from auditory or visual information. This distinction provides a testable criterion for differentiating between pheromone-based and auditory-based models of human sex recognition.

1. According to the HSR model, male and female voices may engage partially distinct neural processing streams within the auditory–limbic system. While this distinction is not yet established anatomically, it is consistent with evidence for sex-differentiated processing of vocal cues and is experimentally testable using neuroimaging approaches. Prior neuroimaging studies have demonstrated sex-differentiated cortical processing of voices and acoustic features: tonotopic maps in auditory cortex (Talavage et al., 2004), hemispheric asymmetries in voice perception (Lattner et al., 2004), sex-specific voice activations in male brains (Sokhi et al., 2005), gender-sensitive cerebral processing of voices (Charest et al., 2012), and distinct cortical representations of pitch and timbre (Allen et al., 2016). Similar approaches could be adapted to test whether male and female voices engage different MGN–hypothalamic pathways, as predicted by the HSR model.

## 6 Discussion

As discussed above, pheromone-based models face important limitations in humans, including the absence of a clearly functional vomeronasal system and inconsistent behavioral evidence. The HSR model synthesizes prenatal auditory sensitivity, infant voice selectivity, cross-modal learning, and pubertal neuroendocrine changes into a coherent account. It suggests that mechanisms underlying sex recognition are prepared developmentally through auditory–visual learning, and may contribute to later processes such as sexual attraction and partner preference. While chemosensory cues may influence adult mood or arousal, they are unlikely to be foundational recognition mechanisms. The HSR framework should be seen as the latest iteration of a theory proposed in 2011 (Salu, 2011) and refined in 2020 (Salu, 2020).

Additional support for the role of auditory cues in the development of sex-related circuits comes from animal studies. For example, work in rodents has shown that early auditory experience can contribute to the formation of neural pathways involved in sexual and social behavior, often in conjunction with olfactory cues (Asaba et al., 2014). These findings suggest that auditory signals can play a developmental role in shaping sex-related neural systems, even in species where chemosensory pathways are prominent.

Within this broader comparative context, the HSR model can be viewed as extending this principle to humans, where auditory cues may assume a more central role in the absence of a clearly functional vomeronasal system. Thus, rather than introducing a fundamentally new mechanism, the model emphasizes a shift in the relative contribution of sensory modalities within a conserved neural architecture.

These findings in rodents also point to a useful direction for future research. In rodents, sex-related behaviors are supported by well-characterized pathways linking sensory input—particularly from the vomeronasal system—to limbic and hypothalamic structures. The demonstration that auditory experience can shape mate preference suggests that auditory signals may access or interact with these same circuits, either through indirect cortico–limbic routes or via more direct subcortical projections.

A more detailed characterization of how auditory inputs interface with limbic–hypothalamic systems in rodents could therefore provide important insights into the organization of analogous pathways in humans. While the dominant sensory modalities differ across species, the underlying circuit architecture is likely to be partially conserved. In this context, comparative studies that map the anatomical and functional connections between auditory pathways and limbic–hypothalamic regions may help identify candidate routes through which auditory signals influence sex recognition in humans. Such work would offer a valuable bridge between well-established animal models and the hypotheses proposed in the HSR framework. This approach may be particularly informative for evaluating whether auditory pathways in humans can functionally substitute for chemosensory inputs observed in other mammals.

Although the HSR model emphasizes general developmental principles, individual differences are expected to influence the acquisition and use of sex recognition cues. For example, reduced auditory input early in life, as in congenital or early-onset hearing impairment, may alter the development of auditory-based recognition and shift greater reliance onto visual or other sensory cues. Variations in the timing of puberty may affect the onset and strength of hypothalamic responsiveness to sex-related stimuli. Neurodevelopmental conditions may influence the integration of auditory and visual information, potentially modifying the formation of cross-modal associations. In addition, cultural and social environments shape the availability and interpretation of learned cues, particularly those related to appearance, behavior, and social roles.

These sources of variability do not contradict the HSR framework but rather define conditions under which its developmental pathways may be altered or expressed differently. As such, they provide opportunities for empirical testing of the model’s predictions regarding the relative contributions of auditory and non-auditory cues across different populations.

One of the strongest arguments for the HSR model is the salience and high fidelity of the human voice as a sex recognition cue. Unlike other sensory modalities, the voice undergoes clear, universal, and biologically timed changes at puberty, precisely when humans reach sexual maturity. This developmental convergence suggests that the voice may function as a primary scaffold for human sex recognition. The timing of these changes further implies the existence of innate pathways—direct or indirect—linking auditory centers such as the medial geniculate nucleus (MGN) to the hypothalamus. Such pathways could provide the neural substrate that guides the development of sex related behavior during childhood and supports its mature expression after puberty.

### 6.1 Biological feasibility

Biological feasibility is an important criterion in evaluating scientific models. Pheromone-based models of human sex recognition face significant challenges here, as the human vomeronasal organ is vestigial (Meredith, 2001) and no universal human sex pheromone has been identified. By contrast, the HSR model is consistent with comparative evidence across vertebrates: in birds, frogs, and other species, auditory cues such as song and call structure are central to sex recognition. These parallels suggest that reliance on voice as a scaffold for sex recognition in humans is not only plausible but evolutionarily parsimonious. Voice changes at puberty, driven by sex hormones, further demonstrate that nature has prepared this cue for recognition of sexually mature individuals. Other innate visual cues may serve as redundant safeguards, but the cross-species evidence strengthens the biological credibility of the HSR framework compared to pheromone-based accounts.

## 7 Conclusion

Human sex recognition can be understood as the outcome of a developmental process in which auditory cues provide an early scaffold for the acquisition of multimodal information, which later becomes integrated within limbic–hypothalamic circuits. By proposing a sensory substitution at the level of input, the HSR model offers a biologically grounded alternative to pheromone-based accounts while preserving their core network architecture. The framework generates testable predictions and provides a structured basis for investigating the development and neural organization of sex recognition in humans.

## Data Availability

The original contributions presented in the study are included in the article/supplementary material, further inquiries can be directed to the corresponding author.
